# Wnt Signaling in Inner Blood–Retinal Barrier Maintenance

**DOI:** 10.3390/ijms222111877

**Published:** 2021-11-02

**Authors:** Felix Yemanyi, Kiran Bora, Alexandra K. Blomfield, Zhongxiao Wang, Jing Chen

**Affiliations:** Department of Ophthalmology, Boston Children’s Hospital, Harvard Medical School, Boston, MA 02115, USA; Felix.Yemanyi@childrens.harvard.edu (F.Y.); Kiran.Bora@childrens.harvard.edu (K.B.); Alexandra.Blomfield@childrens.harvard.edu (A.K.B.); dr.iris.wang@gmail.com (Z.W.)

**Keywords:** blood–retinal barrier, Wnt signaling, endothelial cells, paracellular transport, transcytosis

## Abstract

The retina is a light-sensing ocular tissue that sends information to the brain to enable vision. The blood–retinal barrier (BRB) contributes to maintaining homeostasis in the retinal microenvironment by selectively regulating flux of molecules between systemic circulation and the retina. Maintaining such physiological balance is fundamental to visual function by facilitating the delivery of nutrients and oxygen and for protection from blood-borne toxins. The inner BRB (iBRB), composed mostly of inner retinal vasculature, controls substance exchange mainly via transportation processes between (paracellular) and through (transcellular) the retinal microvascular endothelium. Disruption of iBRB, characterized by retinal edema, is observed in many eye diseases and disturbs the physiological quiescence in the retina’s extracellular space, resulting in vision loss. Consequently, understanding the mechanisms of iBRB formation, maintenance, and breakdown is pivotal to discovering potential targets to restore function to compromised physiological barriers. These unraveled targets can also inform potential drug delivery strategies across the BRB and the blood–brain barrier into retinas and brain tissues, respectively. This review summarizes mechanistic insights into the development and maintenance of iBRB in health and disease, with a specific focus on the Wnt signaling pathway and its regulatory role in both paracellular and transcellular transport across the retinal vascular endothelium.

## 1. Introduction

The retina, an extension of the brain and part of the central nervous system (CNS), plays an important function in converting photons into electrochemical signals to allow visual perception. Per tissue weight, the retina is one of the most metabolically active and demanding tissues in the body [1]. This high metabolic demand requires mechanisms to facilitate the effective and efficient supply of nutrients and oxygen from the circulation, prompt the removal of metabolic waste, and protect from blood-borne toxins and pathogens. The functional exchange of metabolic substances between the retina and the circulation depends on a stable neurovascular microenvironment, which is also important for light transmission and visual function. Such physiological balance in the retina’s extracellular space is maintained and regulated in part by the blood–retinal barrier (BRB).

The existence of BRB was first observed by Schnaudigel [2] in 1913 by showing the CNS and retinal barrier properties resistant to trypan blue dye in rabbits, and was later studied in more detail by Palm [3] in 1947 by comparing the barriers in the retina and the brain. With similar dye approaches, in 1966 Cunha-Vaz and colleagues [4] suggested the retinal vascular endothelium as the barrier site in several species including cats, rabbits, and rats. Similar to the function of the blood–brain barrier (BBB) in the brain [5], the BRB is a restrictive physiological barrier that regulates ion, protein, cell, and water flux from the retinal and choroidal vasculatures into and out of the retina. The BRB primarily comprises two continuous monolayers of cells with a distinct spatial localization and structure: the retinal pigment epithelium (RPE) as the major component of the outer BRB (oBRB), and the retinal microvascular endothelial cell (RMEC) as the core constituent of the inner BRB (iBRB) [6,7].

Dysfunction of the BRB is implicated in several debilitating, blinding retinal diseases wherein impaired BRB disturbs the stability of the retinal microenvironment with adverse effects on nutrient and oxygen supply, waste removal, and light absorption. For instance, loss of the iBRB is implicated in diabetic retinopathy (DR), retinopathy of prematurity (ROP), retinal vein occlusion, retinitis pigmentosa, and retinoblastoma [8,9,10,11]. These eye diseases are the leading causes of vision impairment affecting both adults and children worldwide. On the other hand, impaired oBRB is observed in age-related macular degeneration (AMD) [6,12], a major cause of vision loss in the elderly. Therefore, the importance of discovering novel therapies that restore function to compromised BRB cannot be overemphasized. However, to develop such therapies, a better understanding of the regulatory mechanisms that underpin BRB development, maintenance, and disruption is crucial. Over the past two decades, substantial insights into iBRB development have emerged from studies on rare vascular eye diseases including familial exudative vitreoretinopathy (FEVR) and Norrie disease, both of which are linked with mutations in the Wnt signaling pathway [13,14,15,16]. Studies on animal models of FEVR and Norrie disease have greatly shaped our current understanding of the key function of the Wnt signaling pathway in regulating BRB through both paracellular and transcellular transport across RMECs [9,17,18,19,20].

This review focuses on the role of Wnt signaling in maintaining iBRB. The cellular and molecular composition of the iBRB together with its development is introduced first, followed by a summary of essential knowledge on the mechanistic foundations of Wnt signaling in iBRB function. Other crucial mechanisms of iBRB maintenance and breakdown in health and disease are also briefly discussed with their relevance to Wnt signaling. Finally, we postulate a possible future inquiry into the role of the Wnt signaling pathway in regulating ocular barriergenesis and the possibility of targeting this pathway as a therapeutic intervention to improve BRB function.

## 2. Molecular Components of the iBRB

### 2.1. Retinal Vascular Endothelium Is the Cellular Site of iBRB

**Figure 1:**
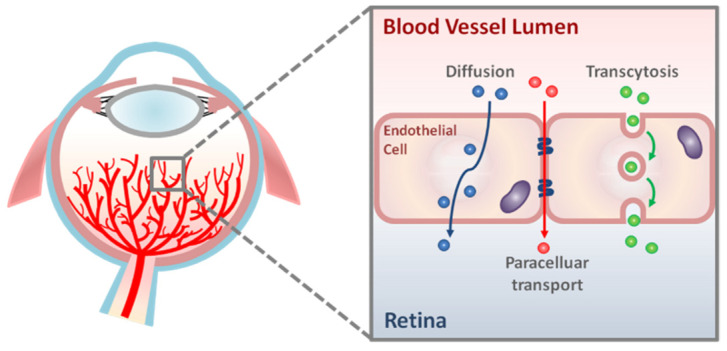
**Schematic representation of transport pathways across the retinal vascular endothelium.** There are two main routes of molecular transport across retinal vascular endothelial cells: paracellular transport and transcellular transport (diffusion and transcytosis).

**Figure 2:**
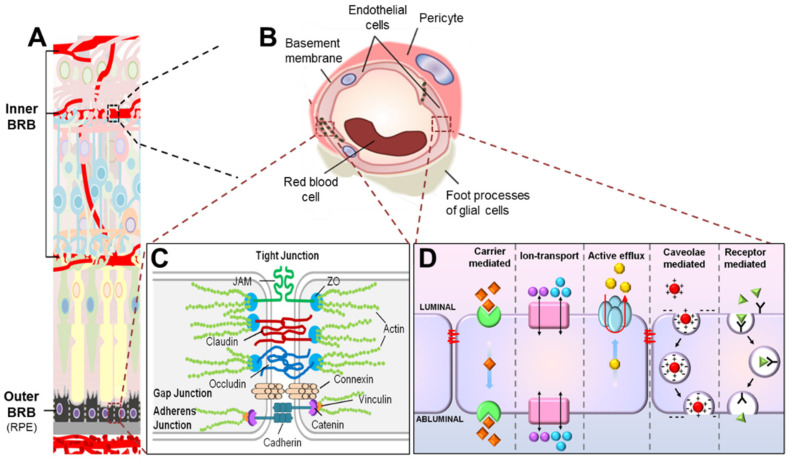
**Scheme illustrating composition of the blood–retinal barrier (BRB).** (**A**) The BRB consists of two parts, the inner BRB (iBRB) and the outer BRB (oBRB), that together regulate exchange of substances between the retina and the systemic circulation. The retinal microvasculature is the primary component of the iBRB, whereas the retinal pigment epithelium (RPE) mainly forms the oBRB. (**B**) Magnified cross-sectional view of the retinal vessel showing constituents of the iBRB. The iBRB is formed by both cellular and non-cellular components of the neurovascular unit including retinal microvascular endothelial cells (RMECs), basement membranes, pericytes, and end-foot processes of glial cells including astrocytes and Müller glia. (**C**) Transport of substances between the systemic circulation and the inner retina between RMECs (paracellular transport) is tightly regulated by junctional protein complexes including tight junctions, adherens junctions, and gap junctions. (**D**) On the other hand, substance exchange between the systemic circulation and the inner retina through RMECs (transcellular transport) is partly mediated by energy-dependent transport systems including carrier-mediated transport, ion transport, active efflux transport, caveolae-mediated transport, and receptor-mediated transport. JAM, junction adhesion molecule; ZO, zona occludens. Figure adapted with permission from Chen et al., *Anti-Angiogenic Therapy in Ophthalmology*, 1–19, Springer International Publishing, 2016.

Molecular flux across the iBRB is primarily regulated by a network of well-organized retinal vasculature and RMECs lining the lumen of these vessels (Figure 1 and Figure 2). Microvascular endothelial cells (ECs) in the CNS, including the retina, have a specialized barrier property that differs from that of the endothelium in peripheral tissues elsewhere in the body. This barrier property in RMECs is achieved by a continuous array of intercellular tight junctions without any fenestrations, and also by the profoundly low rates of transcytosis [21]. Together, these two features of cellular specialization substantially limit both paracellular and transcellular movement of molecules across RMECs under physiological conditions. As a result, the exchange of substances across RMECs is often controlled by a series of specific junctional proteins and transporters. In addition, the barrier property of RMECs is also maintained in part by their crosstalk with other cellular and non-cellular components of the neurovascular unit, including pericytes, smooth muscle cells, Müller glia, astrocytes, inner basal lamina (shared by endothelial cells and pericytes), and outer basal lamina (produced by glial cells) [22] (Figure 2B). Together, they allow two main forms of solute and fluid movement across RMECs: paracellular transport (‘between’ cells via tight junctions) and transcellular transport (‘through’ cells; by passive diffusion or active transcytosis) (Figure 1). 

### 2.2. Endothelial Junctions Are Key Components of Paracellular Transport across the iBRB

Water and small, water-soluble compounds (typically smaller than 3 nm in radius and <500 Da [23]) can pass across the iBRB via paracellular transport, regulated by the dynamic opening and closing of sophisticated junctional protein complexes connecting and sealing two adjacent RMECs (Figure 2B). These protein units consist of several functional groups of junctions, including tight junctions, adherens junctions, and gap junctions (Figure 2C). Together, they regulate cell-to-cell adhesion and contact inhibition of EC growth, maintaining cell polarity and cell survival and ultimately modulating paracellular permeability [7,24,25]. Unlike epithelial cells, which normally have each group of their junctions as separate entities, EC junctions are frequently entangled with one another to form even more complex junctional proteins [7,26]. For instance, in RMECs, tight junctions are usually intertwined with adherens junctions and gap junctions to form higher structural protein complexes consistent with their function in selectively restricting paracellular transport [7].

#### 2.2.1. Tight Junctions Are Mainly Composed of ZO, Occludin, and Claudins

‘Zonula Occludens’ 1 (ZO-1) was the first protein discovered to be associated with tight junctions [27], followed by ZO-2 (tight junction protein 2) and ZO-3 (tight junction protein 3) [28,29]. ZOs are adaptor or scaffolding proteins because they help anchor cytoplasmic tails of tight junctional proteins (for example, cingulin) to the actin cytoskeleton [30]. However, the first tight junctional protein to be implicated in regulating paracellular transport was occludin, which has four transmembrane domains [31]. Later, the respective formation of tight junctions in cells or mice, despite suppression or deficiency of occludin, motivated the discovery of claudins as a new family of tight junctional proteins [32]. 

There are 27 family members of claudins, and each one can form complex structures with one another or with occludin [32,33]. In brain vessels, 12 claudins have been identified as components of the BBB: claudin1, 3, 5, 8, 10, 12, 15, 17, 19, 20, 22, and 23 [34]. On the other hand, in the mouse retina, 17 claudins have been identified (claudin1, 2, 3, 4, 5, 7, 9, 10, 11, 12, 13, 14, 17, 19, 20, 22, and 23). Among claudin1, 2, 3, and 5, all have been identified as tight junction components; claudin5 is the most abundantly expressed in both the retina and brain vessels [8,24,34,35]. Other molecular components of endothelial tight junctional proteins include the vascular endothelial cell-specific adhesion molecule, poliovirus receptor-related-1 (also known as nectin-1) and junctional adhesion molecules (JAMs), and the coxsackievirus and adenovirus receptor (CAR), all of which belong to immunoglobulin-like superfamily molecules. The JAM family proteins consist of members such as JAM-A, JAM-B, JAM-C, JAM-like (JAML), and JAM4, where JAM-A and JAM-C have been identified as critical components of RMEC tight junctions [7,36].

#### 2.2.2. Formation of Tight Junction Is Facilitated by Adherens Junctions and Gap Junctions

The formation of tight junctions between RMECs is preceded and stimulated by adherens junctions, which enhance intercellular contact during early embryonic development [37]. For instance, vascular endothelial cadherin (VE-cadherin), a major constituent of endothelial adherens junctions, induces the expression of claudin5 [38]. Therefore, modulations in the adhesive properties of VE-cadherin could regulate the barrier function of RMECs by stabilizing tight junctions and/or controlling their dynamic opening or closing. Multiple intracellular molecules such as β-catenin, p120, plakoglobin, density-enhanced phosphatase 1, and vascular endothelial protein tyrosine phosphatase bind VE-cadherin [39] could possibly have a role in mediating VE-cadherin-dependent changes in tight junctions. Neural (N)-cadherin, with a more prominent role in cell adhesion in cadiomyocytes and neuronal synapses, is another abundant cadherin identified in the adherens junctions in HRMECs [7].

The assembly of adherens junctions and tight junctions is facilitated by gap junctions [40], suggesting that gap junctions could be indispensable to paracellular transport regulation and inner retinal barriergenesis. Gap junctions consist of a hemi-channel (or connexon) on each adjacent cell, which is formed by six identical or different connexins (Cx). Gap junctions enhance electrical and chemical communication between cells, permitting the free movement of small molecules (<1 kDa) [7]. In the retina or brain, gap junctions are found mainly in astrocytes [41] but are also located between adjacent microvascular ECs or between microvascular ECs and pericytes. In the retina, and particularly in RMECs, Cx7, Cx40, and Cx43 are widely expressed and could have important roles in barriergenesis [42].

To summarize, the junctional proteins of RMECs do not function in isolation but are structurally and functionally interlinked with one another to ensure the precise regulation of paracellular transport in maintaining iBRB integrity.

### 2.3. Transcytosis Is a Main Route of Transcellular Transport across the Inner BRB

Generally, the movement of solute or fluid ‘through’ RMECs in the inner retina is tightly regulated by energy-dependent membrane transporters and vesicular transport. The exception is a wide range of dissolved or gaseous lipid-soluble molecules, such as oxygen, where passive transport across RMECs occurs via diffusion following a concentration gradient. All other types of energy-mediated transcellular transport across RMECs can be grouped into five main categories: carrier-mediated transport, ion transport, active efflux transport, receptor-mediated transport, and caveolae-mediated transport (Figure 2D). For instance, carrier-mediated transport systems enhance the influx of nutrients such as glucose, lactate, certain amino acids, and vitamins across ECs [43,44,45]. The ion transport systems mediate ion flux across RMECs and comprise sodium pump (Na^+^, K^+^/ATPase), sodium-potassium-two chloride (Na^+^/K^+^/2Cl^−^) cotransporter, sodium–hydrogen exchanger, chloride–bicarbonate exchanger, and sodium–calcium exchanger [46,47,48]. The active efflux transport systems boost the extrusion of molecules from neural tissues into systemic circulation and include ATP-binding cassette efflux transporters and certain solute carrier transporters [49]. The receptor-mediated transport systems facilitate the transport of neuroactive peptides and large proteins (for example, transferrin and immunoglobulin G) across vascular endothelium [50,51,52,53,54,55,56]. The caveolar-mediated transport encompasses caveolar membranes harboring receptors for the movement of large molecules, such as insulin and albumin, across vascular ECs. The receptor-mediated transport systems and caveolar-mediated transport may overlap sometimes when the receptors are located on a caveolar membrane. A more comprehensive review of each transport category has been previously documented [7,57,58]. Here, we will focus on summarizing the caveolae-dependent vesicle transport (transcytosis).

#### 2.3.1. Caveolin1 Is Essential for Caveolar-Mediated Endothelial Transcytosis

The idea of endothelial transcytosis first emerged in the 1950s in studies by George Palade on vascular permeability, in which small vesicles near capillary plasma membrane were described to explain microvessel permeability [59]. Later, in 1979, Nicolae Simionescu [60] introduced the term transcytosis, defined as the transport of large molecules across ECs from the vascular lumen to the abluminal side by specialized plasmalemmal vesicles. Transcytosis may occur through three different vesicle types: clathrin-dependent endocytosis, clathrin-independent macropinocytosis, and caveolin-assisted caveola uptake [61]. These three types of vesicles have different sizes: the macropinosomes are the largest, averaging 200 to 500 nm; clathrin-coated vesicles are typically between 70 and 150 nm; caveolar vesicles are the smallest, ranging from 50 to 100 nm [61]. Caveolae (meaning little caves) are spherical cell surface plasma membrane invaginations and are a special type of lipid raft [62]. They not only have functions in regulating transcytosis, but are also important in endocytosis, signal transduction, cholesterol homeostasis, and mechanotransduction [57]. 

The core protein components of caveolae are caveolins (Cav) and cavins. There are three Cav (Cav13) and four cavin (Cavin14) proteins [63,64]. Among the three Cav proteins identified, Cav1 is exclusively involved in endothelial caveolae and expressed in the cardiovascular system, whereas Cav3 is muscle-specific [63,65]. Cav2, a truncated form of Cav1, is not essential for caveolae formation, but may assist in caveolae assembly when co-expressed with Cav1 [61]. *Cav1*^−/−^ mice are resistant to drug-induced pulmonary vascular albumin hyperpermeability and edema [66], suggesting a critical role of Cav1-coated caveola in vascular permeability. In the retina, Cav1 is expressed in the developing and mature blood vessels [67], and also in other cell types including neurons and Müller glia [68]. Whereas Cav1 is linked with actin cytoskeleton for vesicle movement, the recruitment of dynamin is also required, which is concentrated at the membrane invagination and is essential for vesicle scission (that is, pinching off of vesicles from the plasma membrane) [61]. The trafficking of endocytosed vesicles, on the other hand, relies on Rab proteins, a group of monomeric GTPases, which assist in vesicle docking and fusion with target membranes (exocytosis) [61].

#### 2.3.2. Normal Mature RMECs Have Low Rate of Caveolar-Mediated Transcytosis

Transcytosis may assist the migration of macromolecules, including albumin, transferrin, insulin, lipoproteins, and possibly immunoglobulins, from the circulation into the retina through their respective receptors located on vesicles. However, under normal physiological conditions, transcytosis is significantly limited in the retina compared with other tissues or organs in order to maintain its barrier property [69,70]. This is achieved in barrier ECs (for instance, RMECs) by relatively low numbers of caveolae at their luminal membrane, and by the reduced expression of Cav1, albumin receptors, and other molecules relative to non-barrier ECs. In addition, under normal circumstances, transcytosis appears to favor the enhanced movement of solute and fluid from the retina into the circulation, and not the other way round. For example, the vesicular transport of albumin from the retina into the circulation is higher than the reverse, as demonstrated by higher vesicle density at the abluminal side of retinal blood vessels [71]. Although the specific directional regulation of albumin transport remains unknown, it has the potential to regulate protein gradients concomitant with the movement of fluids in the retina. Intriguingly, mice with genetic deficiency of Cav1 show changes in the expression of tight junctional protein claudin5 [67], consistent with previous studies on brain microvascular ECs [72,73]. These observations suggest that caveolin-mediated transcytosis may be associated with the regulation of paracellular transport, or potentially reflect a secondary compensatory change in junctional proteins in the absence of Cav1. 

#### 2.3.3. Protein Markers of High and Low EC Transcytosis: PLVAP and MFSD2A

In the developing retina, functional iBRB integrity is acquired in both spatial and temporal manner following the developmental reduction in endothelial transcytosis [74], which has hitherto contributed to the leakage in immature vessels. A common feature in non-barrier endothelial caveolae is the stomatal diaphragm, thin protein structures that form on top of caveolar flasks [75]. A major component of these diaphragms is plasmalemma vesicle-associated protein (PLVAP), which has been implicated in the loss of barrier integrity in several retinal diseases such as DR and ROP. Hence, PLVAP is designated as an EC-specific marker for increased transcytosis [76,77]. 

Another molecule implicated in regulating transcytosis in the CNS is the major facilitator superfamily domain-containing 2a (MFSD2A). MFSD2A, selectively expressed in both brain and retinal microvessels, is a protein with dual roles in both lipid transport and transcytosis. MFSD2A assists brain uptake of docosahexaenoic acid (DHA) (an omega-3 polyunsaturated fatty acid, which is important for brain growth and function) in a form bound with lysophosphatidylcholine (LPC) [78,79]. In the eye, MFSD2A is also linked with an uptake of DHA in photoreceptors through transportation by the RPE in mice [80]. On the other hand, MFSD2A was found to be important for BBB formation and function [81]. Subsequent work showed that the function of MFSD2A in increasing LPC-DHA uptake to control lipid composition in the brain is closely correlated with the reduction in caveolae microdomains in the vascular endothelium to significantly reduce transcytosis independent of tight junctions [82]. These observations indicate that in the brain and retina, MFSD2A is essential for maintaining the barrier integrity of vascular ECs under physiological conditions, in part through its lipid transport role. Therefore, targeted inhibition of MFSD2A has been suggested as a potential route to temporarily disinhibit EC transcytosis across the BBB to facilitate drug delivery [83].

To summarize, under physiological conditions, transcellular-regulated transport appears to be the preferred route for the active transport of macromolecules facilitated by caveolae- and other receptor-mediated transport mechanisms across ECs in BBB, and similarly in RMECs that constitute the iBRB.

## 3. Development of the Inner BRB

As retinal vessel growth and barrier formation are closely associated [84], we will first briefly summarize the spatial and temporal development of retinal vessels, and then outline that of the associated iBRB formation. 

### 3.1. Development of Retinal Vasculature

**Figure 3:**
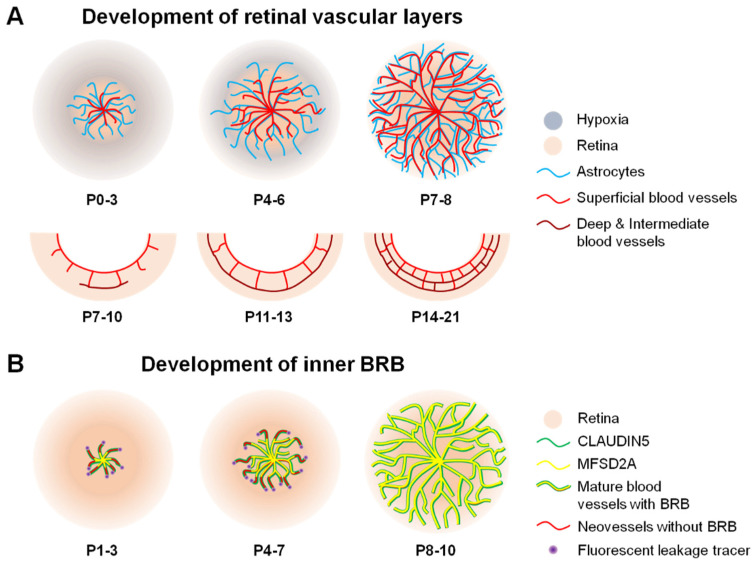
**Scheme illustrating formation of blood vessels and inner BRB (iBRB) in the mouse retina.** (**A**) In mice, vascular sprouting or angiogenesis develops after birth from postnatal day (P) 0. The retinal vessels (red) originate from the optic nerve head after birth, grow radially towards the periphery, and reach the edge of the retina around P7-8. Astrocytes (blue) precede and serve as a template for the newly developing retinal vessels. Superficial vascular plexus follows a hypothesized physiological hypoxia wave (grey) and astrocyte template (blue). Complete formation of superficial vasculature is followed by development of deep vessel formation during the second week of birth and subsequent formation of an intermediate vascular layer. The whole retinal vasculature growth takes approximately three weeks after birth in mice. In humans, this process occurs prenatally. (**B**) Formation of the iBRB proceeds in a central-to-peripheral pattern in the retina, beginning with the presence of tight junctional protein claudin5 (green) as early as P1, which matures by P3 to cover the entire length of newly formed vessels in mice. However, these new sprouting neovessels are still leaky (red) and injected fluorescent leakage tracers (purple) can be detected around neovessels. From P3 onward, the full length of these vessels starts to be lined with the transcytosis modulating protein, MFSD2A (yellow). This process continues through P10, at which point the superficial retinal vasculature has completely formed and matured along with the BRB. BRB, blood–retinal barrier; MFSD2A, major facilitator superfamily domain-containing protein 2a. Figure adapted with permission from Chen et al., *Anti-Angiogenic Therapy in Ophthalmology*, 1–19, Springer International Publishing, 2016.

In early human and rodent embryonic eye development, a transient hyaloid network of vasculature emerges from the central hyaloid artery in the optic nerve, providing vascular support for the developing lens and retina. Hyaloid vessels begin to regress around gestation week (GW) 14 in humans and after birth in rodents, concurrent with the development of new retinal vasculature [85,86]. Astrocytes typically precede and suggested to serve as a template for the development of this new retinal vasculature in mice. Astrocyte precursor cells emerge from the optic nerve head, migrate towards the ganglion cell layer, and begin to differentiate into astrocytes, and can be detected as early as GW14 in humans and postnatal day (P) 0 in mice. Inside the retina, a physiological relative hypoxic wave preceding the vascular network induces astrocytes to secrete vascular endothelial growth factor (VEGF). Coinciding with hyaloid regression in humans, vascular patent cells (VPCs) migrate into the retina and expand through the perifoveal region. VPCs differentiate to form the first vascular plexus layer in a process known as vasculogenesis, which constitutes about 67% of the superficial vascular layer [24,87,88,89,90]. Vasculogenesis has not been observed in rodents. Instead, vascular sprouting or angiogenesis across the retinal ganglion layer (superficial vascular plexus) is observed from P1 and is fully formed by P8 in mice. This process initiates around GW14–16 in humans and continues through GW36–40 when the superficial vasculature reaches retinal periphery. Pericytes are also detected in proximity to endothelial tip cells within the superficial vascular sprouting front starting at around P5 in mice, suggesting their participatory role in retinal vessel formation. Afterwards, ECs sprout into the deep layers of the retina from about P7 to P12 in mice to form the deep vascular layer first, and this deep vascular layer formation is seen at approximately GW21 in humans. Consequently, the intermediate vascular layer is formed last and observed from approximately P14 to P21 in mice and from about GW30 to GW32 in humans [24,88] (Figure 3A). 

### 3.2. Formation of iBRB

Much of our understanding on iBRB formation comes from studies on mice (Figure 3B). The development of the iBRB occurs from P1 to P10 in mice, concurrent with complete superficial retinal vessel formation. Similar to retinal angiogenesis, iBRB formation is concomitant with the presence of astrocytes at P0. Despite an apparent maturation of the tight junctional protein claudin5 in retinal vascular endothelium from P1 to P3, leakiness in new sprouting retinal vessels (via 3–70 Kd fluorescent and biotin tracers) can still be detected [74]. This vessel leakage reduces in correlation with the complete formation of the transcytosis regulatory protein MFSD2A from P3 to P10 in a ‘central retina to peripheral retina’ fashion [24,74,91]. This observation suggests that retinal barriergenesis matures along the radial growth of retinal vessels, firstly in the central retina towards the peripheral retina, and that the maintenance of iBRB integrity appears to be dependent on the tight regulation of transcellular transport. A functional iBRB is fully developed by P10 in mice, whereas the intermediate layer of the retinal vasculature is yet to be formed [24]. In addition, pericyte recruitment (initiated as early as P5 in mice) and glial processes continuously interact with the vascular endothelium through P16 to further strengthen iBRB integrity just as has been demonstrated for BBB maintenance, where barrier integrity is inversely correlated with pericyte coverage [24,34,74,84,91,92,93]. Intriguingly, the ratio of pericytes to endothelial cells in the iBRB (1:1) is higher than the BBB (1:3) [94], probably indicating a greater requirement for pericytes in the retina to maintain barrier integrity compared with the brain. This difference in the pericyte–endothelial cell ratio in the retina vs. brain may also reflect discrepancies in their vessel composition; for example, compared with the retina, the brain has a greater number of large vessels (that is, arteries and veins) that typically lack pericytes, which mostly wrap around capillaries. 

Together, not only is the maturation of cells, including RMECs, in the neurovascular unit important for physiological retinal vascularization, but also fundamental to iBRB development and maintenance. In the mouse, an intact iBRB is established early on while the retinal vasculature is still under development and before eye opening [24], suggesting the importance of functional iBRB in retinal vascular and neuronal development.

## 4. Wnt Signaling and iBRB Maintenance

The formation of blood vessels and barrier maturation in the eye are tightly coordinated, although the underlying mechanisms regulating angiogenesis vs. barriergenesis are different. For instance, VEGF is a well-known endothelial growth factor, yet it promotes the breakdown of vascular barriergenesis by inducing permeability; it was formerly named Vascular Permeability Factors when originally discovered [95]. Accordingly, following the inhibition of neural activity that induces delayed developmental angiogenesis in mice, exogenous VEGF restores vessel growth but not iBRB function, whereas the stabilizing of β-catenin (a Wnt signaling effector) in ECs rescues BRB dysfunction but not vessel formation [96]. In addition, the EC-specific deletion of SRY-box transcription factor 7 (Sox7), Sox17, and Sox18, which are all genes modulated by canonical Wnt signaling, leads to profound retinal edema, despite the nearly normal retinal vascular morphology in adult mice [97]. 

The Wnt signaling pathway is crucial to developmental and pathological angiogenesis in many organs and tissues, including the eye and retina [16,98,99,100,101]. Emerging evidence also suggests its regulatory role in CNS and retinal barrier formation and maturation [5,15,102]. An experimental inquiry into Wnt signaling-dependent iBRB regulation could not be timelier and more appropriate given that current therapies (for example, anti-VEGFs) for restoring barriergenesis in retinal vascular diseases have limitations in their efficacy [103]. In addition, studies on the mechanisms underlying the Wnt signaling-mediated control of retinal barriergenesis may provide new opportunities to overcome tissue and cellular hindrances to drug delivery, especially in the CNS. Before we delve into how the Wnt signaling pathway mediates iBRB in retinal health and disease, here we render a brief description of its signaling components. A more comprehensive review of Wnt signaling pathway constituents has been previously documented [16,104].

### 4.1. Molecular Components of the Wnt Signaling Pathway

The Wnt signaling pathway consists of two main types: the canonical Wnt/β-catenin-dependent and the non-canonical Wnt/β-catenin-independent pathways. Although the latter has not been as well characterized as the former, examples of the non-canonical ligands include Wnt5a and Wnt11 (there are 19 secreted, cysteine-rich Wnt ligands [105]), with the calcium and planar cell polarity pathways characterized as probable downstream signaling targets. The non-canonical Wnt signaling pathway has been reported to be important in vascular development and/or remodeling in response to shear stress or by regulating VEGF availability [106,107]. Additionally, it may inhibit the canonical Wnt/β-catenin signaling pathway in a ligand- or receptor-mediated manner [108,109]. On the other hand, the relatively well-studied canonical Wnt/β-catenin signaling pathway consists of canonical Wnt ligands such as Wnt1, 3, 5b, 7b, 10a, 13 (Wnt2b) and the unconventional Wnt ligand Norrin, which is a transforming growth factor beta (TGFβ) family member produced by Müller glia and astrocytes [110] and also found in macrophages [9] and ECs [111]. 

**Figure 4:**
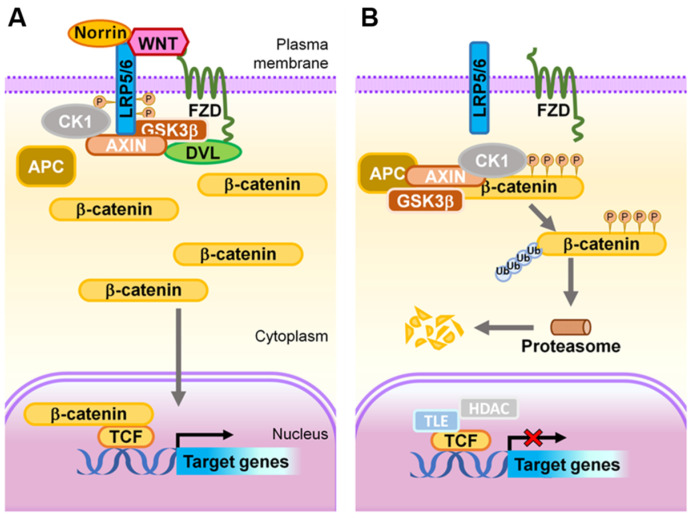
**Schematic representation of the Wnt signaling transduction cascade.** (**A**) ON state: In the presence of Wnt ligands or Norrin, LRP5/6 is recruited into the receptor complex with Wnt- or Norrin-bound FZD, which further recruits DVL proteins, resulting in subsequent phosphorylation of LRP5/6 by GSK3 and CK1, and Axin on-boarding. The destruction of APC/Axin/GSK3β complex prevents phosphorylation of β-catenin, hence allowing its stabilization and accumulation within the cytoplasm. The stabilized β-catenin subsequently translocates into the nucleus where it binds to LEF/TCF transcription factors to transactivate expression of Wnt target genes. (**B**) OFF state: In the absence of a Wnt ligand, or when it is inhibited from binding to FZD and LRP5/6, the APC/Axin/GSK3β complex and CK1 synergistically target cytoplasmic β-catenin for phosphorylation, ubiquitination, and subsequent proteasomal degradation. As a result, in this ‘off’ state, transcription of Wnt signaling responsive genes is attenuated by TCF-TLE1/Groucho and HDAC. APC, adenomatous polyposis coli; CK1, casein kinase 1; DVL, Dishevelled; FZD, Frizzled; GSK3, glycogen synthase kinase 3; HDAC, histone deacetylases; LRP, low-density lipoprotein receptor-related protein; TCF, T cell factor; TLE1, transducing-like enhancer of split-1. Figure adapted with permission from Wang, et al., *Prog Retin Eye Res*. 2019.

Activation of canonical Wnt signaling to the ‘on’ state starts with Wnt ligands binding to one of several Frizzled (Fzd) family receptors (there are 10 Frizzled receptors in vertebrates and Fzd4 is expressed in endothelial cells), typically in addition to a co-receptor, such as low-density lipoprotein receptor-related protein 5 (LRP5) or LRP6 (Figure 4A), to activate downstream intracellular signaling mediators. Further, signal specificity is often regulated by the availability of compulsory co-receptors, such as GRP124/Reck for Wnt7a/b [112,113,114] and Tspan12 for Norrin [115]. This leads to the phosphorylation and activation of Dishevelled (Dvl) proteins (Dvl1–3). Phosphorylated Dvl then recruits Axin to the cell membrane, and subsequently induces the degradation of Axin to inhibit glycogen synthase kinase 3β (GSK3β)-dependent phosphorylation of β-catenin. In the absence of any phosphorylation, β-catenin stabilizes in the cytoplasm and subsequently translocates into the nucleus where it binds to lymphoid enhancer factor/T-cell factor (LEF/TCF) transcription factors. This results in the activation of Wnt target genes, including the so-called Yamanaka factors important for embryonic stem cells and for inducing cellular pluripotency, c-Myc [116], Oct4 [117], Sox2 [118], the critical cell cycle regulator cyclin D [119], and angiogenic genes such as VEGF [120] and VEGFR2 [121], and the intercellular adhesion molecule 1 (ICAM1) [122], twist [123], and jagged1 [124]. These observations demonstrate the diverse roles of the Wnt signaling pathway in various biological processes in many organs beyond the eye. 

Conversely, in the absence of Wnt ligands (inactive ‘off’ state), β-catenin is recruited to a destruction complex consisting of Axin, adenomatous polyposis coli (APC), casein kinase 1α (CK1α), and GSK3β. β-catenin is sequentially phosphorylated by CK1α and GSK3β to create a binding site for the ubiquitin E3-ligase βTrCP, which targets β-catenin for ubiquitination and proteasomal degradation in the cytoplasm [104], disabling nuclear translocation and the activation of target genes (Figure 4B). It is worth noting that in addition to the transcriptional signaling role of β-catenin, this also plays a separate role as a structural component of an adherens junction by binding to cadherin and α-catenin, and then actin cytoskeleton (described in Section 2.2). Whether there are two distinct pools of β-catenin for these two separate functions or whether the same β-catenin molecule can shuffle between the two roles is not entirely clear yet. However, there is evidence that VE-cadherin upregulates the expression of claudin5 in part by preventing the nuclear accumulation of β-catenin [38,125].

### 4.2. Wnt Signaling Pathway Restricts Paracellular Transport in iBRB

Much of our understanding of Wnt signaling in retinal angiogenesis comes from experimental studies of two rare, congenital, potentially blinding eye diseases: X-linked Norrie disease and FEVR, which are linked with Wnt signaling mutations in humans in either receptors (FZD4, LRP5, TSPAN12) or the ligand Norrin [15,16], respectively. Hence, *Norrin*-, *Fzd4*-, *Lrp5*-, and *Tspan12*-deficient mice, all of which exhibit delayed developmental angiogenesis and model these two rare human congenital eye diseases, are emerging as experimental models for studying not only retinal angiogenesis but also ocular barriergenesis due to their compromised BRB [17,115,126,127]. There is strong evidence supporting the notion that canonical Wnt signaling-mediated vascular development goes hand in hand with specification of the iBRB or BBB. For instance, mice with targeted systemic deletion in LRP5, Norrin, and FZD4 or EC-specific deletion of LRP6, not only had vascular developmental defects in the retina and brain but also lost plasticity of BRB and BBB properties, which were ameliorated by stabilizing the β-catenin [18]. These observations suggest the key role of canonical β-catenin signaling in both angiogenesis and barriergenesis. 

The idea that Wnt/β-catenin signaling maintains iBRB, in part through restricting paracellular transport in vascular endothelium, has been supported by multiple studies. This is probably partly due to the fact that β-catenin binds VE-cadherin, which is a prerequisite for tight junction formation [38], suggesting that any changes in Wnt/β-catenin signaling could directly or indirectly modulate tight junctions to regulate paracellular transport. For example, we previously showed that the loss of LRP5 and the downstream signaling molecule Dvl2 markedly decreased developmental and pathological retinal angiogenesis in mice [9] and significantly downregulated the tight junctional protein claudin5 in retinal vessels [99], indicating a potential iBRB breakdown via enhanced paracellular transport. Retinal suppression of claudin5 was also found in *Norrin*-deficient retinas [128]. Similarly, Jeremy Nathan’s group [18,19] also demonstrated that the Norrin/Fzd4/β-catenin signaling axis is crucial to maintaining iBRB function by modulating the expression of claudin5. Their recent transcriptome study comparing highly permeable vessels in the brain (in circumventricular organs) and the eye (in choroid) further supports the key role of β-catenin in mediating barrier-specific gene expression, including claudin5 [127].

More recent studies have identified additional new players of Wnt signaling that can coordinate its effects on angiogenesis and barrier formation, some of those through claudin5. For example, the loss of Discs large homologue 1 (Dlg1), an intracellular scaffolding protein, in vascular endothelium reproduces retinal vascular defects and the breakdown of BRB and BBB, as seen in Wnt-deficient mice. This finding suggests a new role of Dlg1 in Wnt/β-catenin signaling that appears to be independent of Dlg1′s direct interaction with FZD4 [129]. Similarly, the inactivation of integrin-linked kinase (ILK), a cell–matrix mediator and a newly identified FEVR disease gene, in postnatal ECs results in retinal vascular sprouting defects and impaired iBRB in mice; this suggests a link between ILK-mediated cell–matrix regulation and Wnt signaling in FEVR [130]. A recent chemogenomic screening in human pluripotent stem cell-derived ECs identified the inhibitors of the TGFβ signaling pathway as potent inducers of an endothelium barrier (claudin5) that promote EC barrier resistance and decrease vascular permeability [131]. In contrast, another recent report showed that the loss of TGFβ receptor I (Alk5) inhibits deep retinal vessel layer formation and disrupts barrier property. Whereas the overactivation of Wnt signaling does not rescue the deep layer angiogenic defects in *Alk5*-deficient retina, it does reduce their vascular leakage in part through increasing claudin5, suggesting a potential interaction between TGFβ and Wnt signaling not in retinal angiogenesis but in barrier control [132]. Similarly, the deactivation of adenomatous polyposis coli downregulated 1 (Apcdd1), a membrane-bound glycoprotein and a downstream target and inhibitor of the canonical Wnt pathway, promotes Wnt signaling to regulate physiological barrier maturation. Mazzoni and colleagues [133] showed that mice that overexpress Apcdd1 in retinal ECs have reduced vessel density but increased paracellular barrier permeability, and Apcdd1 mutant ECs precociously form the paracellular component of the barrier by regulating claudin5.

Methods to target Wnt signaling have been explored as potential therapeutics to restore iBRB in both development and diseases. Recently, Chidiac and colleagues [134] developed a bispecific antibody (F4L5.13) to trigger FZD4 and LRP5 proximity such that β-catenin signaling is activated. The stimulation of cultured ECs with the antibody caused the reversal of VEGF-induced permeability, partially by promoting surface expression of the junctional proteins ZO-1, claudin3, and claudin5. Moreover, the treatment of *Tspan12*^−/−^ Wnt-deficient mice with the antibody (from P5 to P20) restored retinal angiogenesis and barrier function (claudin5) [134]. Similarly, the injection of Norrin in diabetic rats restored BRB integrity after additional VEGF-induced permeability by inducing claudin5 and the tight junction complex [135], suggesting the possibility of targeting Wnt ligands, receptors, or downstream pathways as therapeutics to restore iBRB integrity in the developmental stage, or by countering hyperpermeability induced by other factors such as VEGF and potentially TGFβ in pathological conditions. Conversely, several other reports have found that the inhibition of Wnt signaling promotes iBRB under different experimental conditions. Hossain and colleagues [136] showed that blocking LRP1, a lipid transporter and positive regulator of Lrp5/Lrp6, to modulate the Wnt/β-catenin signaling pathway significantly restored iBRB and tight junctional proteins (occludin and ZO-1) in streptozotocin (STZ)-induced diabetic mice. This observation suggests that under pathological conditions, an Lrp1-mediated inhibition of the Wnt signaling pathway restores the physiological regulation of paracellular transport across the iBRB. Moreover, our group previously found that the blockade of claudin5 profoundly attenuated Wnt signaling pathway-dependent angiogenesis in vitro and in vivo [9], indicating that the mediators of iBRB maintenance could also be targeted to ameliorate pathological angiogenesis and barriergenesis. In addition, Liu and colleagues [122] demonstrated that nitrosative stress triggers aberrant Wnt signaling pathway in DR, and that the inhibition of peroxynitrite-induced nitrosative stress in retinal ECs significantly decreases Wnt signaling associated with a markedly reduced ICAM1 expression and the restoration of physiological paracellular transport across retinal ECs.

**Figure 5:**
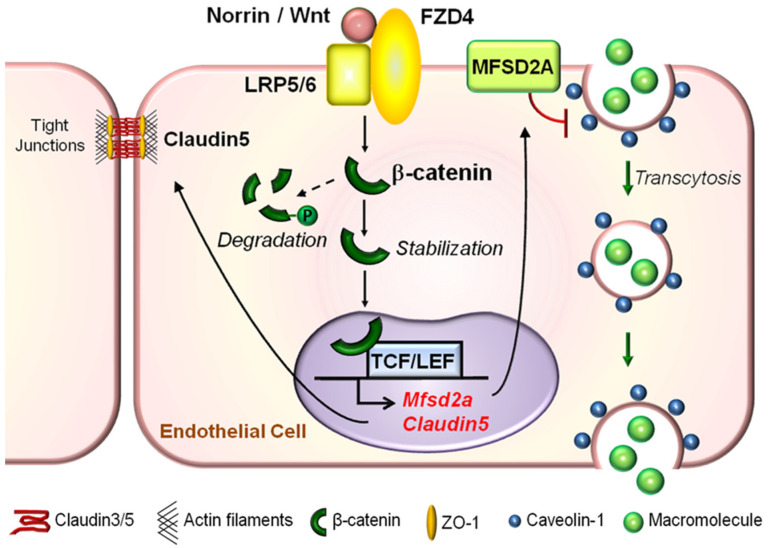
Graphical illustration of molecular mechanisms underlying the role of Wnt signaling in regulating both paracellular transport and transcytosis across the retinal vascular endothelium. Under physiological conditions in the retina, a Wnt ligand (Norrin or Wnt) binds to the FZD4 receptor and LRP5/6 co-receptor complex to activate the canonical Wnt signaling pathway in RMECs. This prevents phosphorylation and degradation of β-catenin in the cytoplasm. Stabilized cytoplasmic β-catenin may either become a major component of adherens junctions to regulate paracellular-mediated iBRB integrity by stabilizing tight junctional proteins such as Claudin5, or translocate into the nucleus to directly bind to and activate expression of *Claudin5* and *Mfsd2a* genes. Whereas the former, Claudin5, is important for maintaining paracellular-dependent iBRB integrity, the latter, MFSD2A, is a membrane transport protein that represses CAV1 protein levels, inhibits CAV1-positive caveolae formation, and restricts EC caveolar transcytosis to maintain iBRB integrity. FZD, Frizzled; LRP, low-density lipoprotein receptor-related protein; TCF, T cell factor; MFSD2A, major facilitator superfamily domain-containing protein 2a; CAV1, caveolin-1; RMEC, retinal microvascular endothelial cells; iBRB, inner blood–retinal barrier; EC, endothelial cells. Figure adapted with permission from Wang et al., *Sci. Adv*. 2020.

Taken together, the canonical Wnt signaling pathway modulates junctional proteins of RMECs, particularly claudin5, in its interaction with other junctional components in paracellular transport across the iBRB in health and disease (Figure 5). Yet, mechanisms of Wnt-mediated iBRB maintenance and breakdown appear to differ between developmental and pathological angiogenesis. The restoration of Wnt signaling is likely to be beneficial when it is downregulated during developmental phases or during non-proliferative stages when vessel dropout occurs to promote angiogenesis and iBRB or to counter hyperpermeability induced by VEGF. However, the suppression of Wnt signaling when it is aberrantly upregulated in proliferative stages of retinopathies may offer likely protection to limit its angiogenic capacity and leakage from pathological neovessels. 

### 4.3. Wnt Signaling Pathway Limits Transcellular Transport in Vascular Endothelium to Maintain Physiological iBRB

The Wnt signaling pathway maintains iBRB not only by tightly regulating the paracellular transport (tight, adherens, and gap junctions) between RMECs, but also by limiting transcellular transport (transmembrane transporters or vesicles) to ensure iBRB stability and integrity. A hint to its link to transcytosis derives from studies on PLVAP, an indicator and marker of EC transcytosis that has an increased expression in both Norrin KO and LRP5 KO mice deficient in Wnt signaling and exhibiting leaky retinal vessels [99,128]. Multiple studies support the key role of the Norrin/Fzd4/LRP5 signaling axis in coordinating angiogenesis and barrier integrity in the retina and brain [101,110,128] associated with dysregulation of PLVAP. For example, Wang and colleagues [19] showed that the gain or loss of Norrin/Fzd4 signaling in the adult retina or cerebellum results in the gain or loss of endothelial cell regulation of iBRB (with increased or decreased PLVAP levels) or BBB function, respectively, consistent with their prior studies [101,110]. One of those studies showed that Sox17, a transcription factor, is a crucial downstream effector of the Norrin/Fzd4 signaling axis in regulating retinal and cerebellar barriergenesis [110]. Later, the same group revealed significant retinal edema in mice only when Sox7, Sox17, and Sox18 were all deleted, and not Sox17 alone, suggesting the functional redundancy of these Wnt signaling-regulated Sox genes [97]. Notwithstanding, together, these observations indicate that Wnt signaling is perpetually required to maintain barriergenesis together with the profound reprogramming of the mature vascular structure in the eye or brain. Subsequently, Zhou and colleagues [18] further demonstrated that the Norrin/Fzd4 signaling-regulated suppression of PLVAP across iBRB or BBB is predominantly dependent on the transcriptional activity of β-catenin. Furthermore, Zhang and colleagues [20] reported that Norrin is a potent trigger of FZD4 ubiquitination and induces the internalization of the Norrin receptor complex into the endo-lysosomal compartment of retinal endothelial cells. They showed that endocytosis of FZD4 in Norrin/Fzd4/β-catenin signaling axis is an important mechanistic step to maintaining iBRB and low PLVAP levels [20]. A more recent study showed that an FZD4/LRP5 agonist fully or partially restored transcellular iBRB function (associated with suppressed PLVAP levels and extravasated FITC dextran) with or without the amelioration of aberrant angiogenesis, respectively, in young (P20) or adult (3-month-old) *Tspan12*^−/−^ Wnt-deficient mouse retinas [134], further supporting the role of PLVAP in iBRB.

Direct evidence of the Wnt signaling pathway mediated modulation of physiological transcytosis in retinal endothelial cells was provided by our recent study [17] where we showed that mice lacking LRP5 or Norrin exhibit increased retinal vascular leakage, and more importantly, exhibit excessive transcytosis across RMECs, as visualized by electron microscopy. To uncover the molecular basis of increased transcytosis following defective Wnt signaling, we first showed that the Wnt signaling pathway directly regulates the transcription of an EC-specific inhibitor of transcytosis, MFSD2A, in a β-catenin-dependent manner. The overexpression of MFSD2A rescues Wnt-deficient-induced transcytosis in ECs and in retinas. Subsequently, we discovered that Wnt signaling mediates MFSD2A-dependent vascular EC transcytosis through a Cav1-positive caveolae pathway independent of clathrin-mediated transport. Furthermore, the levels of omega-3 polyunsaturated fatty acids are decreased in Wnt signaling-deficient retinas, consistent with the fundamental function of MFSD2A as a lipid transporter [79]. Consequently, MFSD2A/Cav1 signaling axis is a novel downstream effector of the Wnt/β-catenin signaling pathway in regulating EC transcytosis and iBRB integrity under physiological conditions and in development [17] (Figure 5). Similar mechanisms of Wnt signaling regulating MFSD2A-dependent caveolar transcytosis may also exist in the brain and BBB, consistent with the reported function of Wnt and MFSD2A/Cav1 in BBB maintenance separately [81,82]. Furthermore, a recent report showed that loss of astrocyte-derived secretion of Wnts induces brain edema, associated with increased brain endothelial Cav-1 vesicles, without alteration in EC junctions [137]. Prior studies have also reported the interaction between caveolar proteins and Wnt signaling components in other tissues. For instance, Cav1 influences Wnt/β-catenin signaling in prostate cancer [138], and in Cav1-deficient stem cells Wnt signaling is upregulated [139,140]. In addition, a direct protein interaction between Cav1 and β-catenin was also reported [141], suggesting Cav1 expression may also inhibit Wnt signaling by recruiting β-catenin to caveolae. Together, these findings suggest the potential bidirectional regulation of Wnt/β-catenin signaling and caveolar-dependent transcytosis.

Whereas the activation of the Wnt signaling pathway may be crucial to maintaining iBRB and BBB during development and under physiological conditions, aberrant or excessive activation of Wnt signaling, as observed in several blinding retinal diseases, may be detrimental to iBRB maintenance. For instance, Yan and colleagues [142] recently determined the probable therapeutic effect of melatonin (an endogenous neurohormone that regulates oxidative stress, inflammation, autophagy, and angiogenesis) on high glucose-induced iBRB in vivo and in vitro, modeling the iBRB breakdown in DR. The authors discovered that melatonin administration ameliorated high glucose-induced iBRB dysfunction via inhibiting the Wnt/β-catenin pathway. Conversely, the protective effect of melatonin on iBRB maintenance under hyperglycemic conditions was reversed by lithium chloride, a potent activator of the Wnt/β-catenin signaling pathway. Similarly, another study in STZ-induced diabetic rats showed significantly reduced retinal extravasated serum albumin (suggesting iBRB restoration) and inflammatory cell infiltration following the inhibition of the Wnt signaling pathway induced by blocking nitrosative stress, a pathological hallmark of DR [122].

In a nutshell, MFSD2A and caveolae are important mechanistic downstream effectors of the canonical Wnt/β-catenin signaling pathway for regulating transcytosis in RMECs under physiological conditions (Figure 5). Whether the upregulation of PLVAP in Wnt-deficient retinas is a secondary consequence reflecting increased transcytosis or a direct transcriptional effect of Wnt signaling remains unclear. Furthermore, a more holistic approach to inform therapeutic strategies will depend on an improved understanding of the detailed modulation of these Wnt signaling-mediated targets and the potential interaction of Wnt signaling with other pathogenic factors in iBRB dysfunction in blinding retinal vascular diseases.

## 5. Interplay of Wnt/β-Catenin Signaling with Other Mechanisms Underpinning iBRB Maintenance and Breakdown in Eye Diseases

The mechanisms of iBRB maintenance and breakdown are clearly multifactorial and transcend the Wnt signaling pathway. For instance, VEGF is a well-known factor that can alter EC junctional proteins to disrupt iBRB integrity in retinal vascular diseases in part through protein kinase C (PKC), and both VEGF and PKC are common drug targets in diabetic retinopathy and diabetic macular edema (DME) [143]. Yet, anti-VEGF therapy has its limitations where some patients remain unresponsive. Thus, it appears combinatory therapeutic approaches beyond VEGF may better resolve retinal vascular leakage or edema in iBRB dysfunction to restore vision in the long run. In this regard, whether Wnt signaling can be targeted independently or in combination with existing approaches to restore iBRB integrity warrants investigation. This section serves only as a brief discussion of other common molecular and cellular mechanisms of iBRB maintenance and breakdown in DR and their potential interplay with Wnt signaling. 

### 5.1. VEGF Is a Main Culprit in DR and DME

DR is one of the most common microvascular complications of diabetes mellitus and a leading cause of vision loss among the working-age population worldwide [144,145]. It shares some mechanistic similarities with other ischemic proliferative retinopathies, including ROP and neovascular AMD, regarding their molecular basis of angiogenic and iBRB control. Diabetic macular edema (DME), partially resulting from iBRB breakdown, is the main cause of vision loss in DR as it can easily be complicated by retinal detachment and vitreous hemorrhage [145]. Mechanisms underlying DME generally consist of altered cellular metabolism [146,147,148,149], inflammation [150,151], oxidative stress [152,153], and extracellular matrix remodeling [154,155,156,157,158,159,160] to mention just a few [7]. These cellular processes often lead to the overexpression of key growth factors [161,162,163,164,165], which subsequently activate several signaling pathways to precipitate iBRB dysfunction and resultant retinal edema [166,167,168,169]. 

Among these growth factors, much work has been carried out on VEGF since the initial discovery that VEGF was upregulated in the ocular fluid of patients with DR and other vascular eye diseases [165]. This finding together with other earlier studies paved the way for anti-VEGF therapy currently used in the clinic for treating AMD, ROP, and DME [170]. This breakthrough for anti-VEGF therapy was partly supported by several experimental studies that demonstrated an overexpression of VEGF and its receptor, VEGFR2 [171,172] an altered cellular metabolism [148], oxidative stress [152,153], inflammation, and growth factors [162,163] in diseased RMECs with compromised paracellular- and transcellular-mediated transport. Critical signaling pathways downstream of the VEGF/VEGFR2 signaling-induced iBRB breakdown are the urokinase plasminogen activator receptor [152,173,174], Src kinase [175], PKCβ [176,177], AKT, extracellular signal-regulated kinase, and c-Jun N-terminal kinase [172]. Despite the success of anti-VEGF therapies, approximately 40% of patients with DME are resistant to anti-VEGF therapy, and for those who do respond, the therapeutic effects do not last long and repeated treatment is required [103]. As a result, Arima and colleagues [178] showed that in diabetic mice, resistance to anti-VEGFs in DME is partly due to inflammation, which activates rho-associated coiled-coil containing kinase 2 to redistribute claudin5. While VEGF is a known Wnt target gene, recent studies on Wnt activation to restore hyperpermeability induced by VEGF provide potential clues on mitigating Wnt signaling to counter iBRB breakdown induced by abnormally high levels of VEGF [134,135]. Yet, much more work is needed to determine in detail the correct time window and level of control needed to titrate Wnt signaling to achieve physiological iBRB control.

### 5.2. Contribution of Other Non-Endothelial Cells in Regulation of iBRB

In addition to the vascular endothelium, aberrant signaling in other components of the neurovascular unit, such as pericytes [179], Mϋller glia, microglia, and astrocytes, also has mechanistic roles in iBRB dysfunction in DR and other retinal vascular diseases. For instance, Notch3 is profoundly downregulated in diabetic mouse pericytes and in hyperglycemia-induced human retinal pericytes, and the suppression of Notch3 in pericytes results in barrier dysfunction of EC monolayers [179]. These findings are consistent with another study in a mouse model of hypertensive retinopathy (HR), a retinal microvascular complication and common ocular presentation of hypertension [180]. In this mouse model of HR, the inactivation of the delta-like ligand 4 (Dll4) or Notch1 disrupted the integrity of iBRB by increasing transcytosis in retinal ECs without any perturbation to junctional protein complexes [181]. However, these observations are in contrast with another study which showed that Notch1 ligands, jagged1, and Dll4 are upregulated in human and mouse diabetic retinal vascular endothelium [182]. Accordingly, the authors demonstrated activated canonical and rapid non-canonical Notch1 pathways that primarily destabilize endothelial cell adherens junctions by causing VE-cadherin to dissociate from β-catenin [182]. Taken together, these research works suggest that the role of Notch signaling in physiological and pathological retinal barriergenesis is cell-, isoform-, and disease-specific. While Wnt and Notch signaling pathways are closely interconnected during embryonic development, whether they also interact during physiological or pathological retinal barriergenesis is not entirely clear and awaits further investigation.

Apart from pericytes, activated microglia can engender increased apoptosis, decreased autophagy, and the increased expression of VEGF in Müller glial cells to destabilize iBRB regulation in DR or under hypoxic conditions [183,184]. They can also upregulate matrix metalloproteinase 9 and downregulate the suppressor of cytokine signaling 3 to activate the Toll-like receptor 4/nuclear factor kappa B signaling axis [185] in iBRB dysfunction. Astrocytes also have their place in iBRB maintenance. A-kinase anchor protein 12 (AKAP12), a scaffolding protein that associates with intracellular molecules, is markedly reduced in the retinas of patients with retinoblastoma, the most common intraocular childhood malignancy, often presenting with iBRB dysfunction [11,186]. A previous study showed that AKAP12 regulates physiological paracellular-mediated barriergenesis (ZO-1 and claudin5) in HRMECs and brain ECs by increasing angiopoietin 1, decreasing VEGF and hypoxia-inducible factor 1α levels, while inhibiting the rho kinase signaling pathway in astrocytes [11,187]. Whether the regulation of barriergenesis genes by AKAP12 is related to or independent of Wnt signaling is still unknown.

Recently, Weiner and colleagues [96] showed that an attenuation of spontaneous cholinergic activity or the repression of starburst amacrine cell numbers hinders the invasion of ECs into the deep layers of the retina and causes iBRB dysfunction in mice. This finding suggests that neural activity from specific neural circuits may also be fundamental to the spatial formation of physiological barriers in the inner retina or even across the CNS. However, the relative contribution and precise relationship between neural activity and critical signaling pathways, such as Wnt signaling, in regulating iBRB integrity remain to be demonstrated. 

To recapitulate, several cellular and non-cellular mechanisms within the retinal neurovascular unit, including retinal ECs or retinal neurons, have been documented to underlie iBRB maintenance and breakdown in health and blinding retinal diseases, respectively. The insufficient efficacy and coverage of existing therapy for ameliorating retinal edema calls for alternative improved therapy or for combination therapy. The latter could be achieved by harnessing the drug development of newly discovered VEGF-independent targets underlying the molecular basis of retinal barriergenesis in health and disease. In this regard, the Wnt signaling pathway appears to be promising in terms of its probable therapeutic efficacy in ameliorating vision loss due to iBRB dysfunction and retinal edema. 

## 6. Conclusions and Future Directions

Research in the past two decades has provided substantial insights into the role of Wnt/β-catenin signaling in not only retinal and CNS angiogenesis but also the regulation of BBB and iBRB. Many genes essential to BBB and iBRB development and maintenance have been discovered as target genes downstream of the Wnt/β-catenin pathway [5]. Claudin5, an essential component of tight junction, is induced by Wnt/β-catenin, which also suppresses PLVAP, a marker of EC transcytosis. High levels of claudin5 and low PLVAP levels have been commonly used as valuable markers of BBB and iBRB integrity in many studies. Our more recent work indicates that Wnt/β-catenin also induces the expression of MFSD2A, to exert the inhibition of EC caveolar transcytosis, maintain transcytosis at low rates under physiological conditions, and suggest a new route of Wnt signaling-mediated regulation of iBRB. These observations have substantial implications for retinal edema where increased transcytosis and the resultant interstitial osmotic pressure have been suggested as major contributors to macular edema and central vision loss [7]. 

Recent works identifying new components of the Wnt/β-catenin signaling pathway (for example, GPR124, Apcdd1 and Dlg1) or potential interactive partners (ILK and TGFβ) have largely expanded our knowledge of BBB and iBRB control related to and beyond Wnt signaling. Uncovering additional players of this pathway, its interaction with other signaling pathways, and its role in regulating the extracellular matrix [188] will potentially open new druggable targets to control CNS and ocular vascular barriergenesis in health and disease. Further, the role of other components of the neurovascular unit, such as astrocyte, pericyte, and microglia, besides RMECs, in regulating Wnt signaling-mediated retinal barriergenesis awaits further inquiry. While most prior works have focused on iBRB and BBB, the regulation of oBRB in the eye, maintained mostly by RPE junctional integrity, has not been studied as extensively. Whether Wnt signaling plays a significant role in regulating RPE barrier integrity remains to be shown. 

Targeting the Wnt signaling pathway to restore BBB and iBRB has been an active area of research. Recent work has demonstrated how Wnt ligand treatment or antibodies activating Wnt or Wnt ligands may restore iBRB breakdown in genetic or diabetic animal models with VEGF-induced hyperpermeability, yet aberrant overactivation of Wnt signaling may underlie iBRB breakdown in other studies. Given the pro-angiogenic effects of the Wnt pathway in pathological angiogenesis, titration of proper levels of Wnt signaling and selection of the optimal treatment window will need to be considered to avoid potential undesirable consequence of stimulating pathological angiogenesis in the proliferative stage of retinopathy. While iBRB and BBB are protective against pathogens and toxins from circulation, they also hinder the delivery of drugs into the CNS and the retina. Finding ways to transiently deactivate the Wnt pathway or suppress its target genes, including *Claudin5* and *Mfsd2a*, and thereby loosening iBRB and BBB to promote drug delivery, will also represent a potential future direction of research [83,189,190].
